# Supplementary material for: MAC Family Transcription Factors Enhance the Tolerance of Mycelia to Heat Stress and Promote the Primordial Formation Rate of Pleurotus ostreatus
Source: J Fungi (Basel). 2023 Dec 27;10(1):13. doi: 10.3390/jof10010013 (PMC10816978; doi:10.3390/jof10010013)
Supplement: Supplementary file 1 [file jof-10-00013-s001.zip › jof-2720573-supplementary.pdf]

Table S1 Primers used in this study.

Primer	Sequence (5′ → 3′)	Note
Po-*gpd*F	GGTACCTTTATTGGCGGT	Promoter cloning
Po-*gpd*R	CCAGGTCAGTGAAATTTCC	
PoMAC1a-F	ATGGTTCTGGTCGACAACAAAAAA	Gene cloning
PoMAC1a-R	CCATTCAGGGATACTTTCCGATAA	
PoMAC1b-F	ATGGTCTTCGTCAATAACAAGAAGT	
PoMAC1b-R	TCAGCGGCCGGCGCAGCAGCTACGT	
PoMAC1a-qF	CAAAACAAAGGTGCCAGACAG	qPCR
PoMAC1a-qR	CATTCACCGCCAGTTTTACAC	
PoMAC1b-qF	CCTATTGAACCAGCTCTTCCC	
PoMAC1b-qR	CATTGGCATCCACGAGTACT	
β-tubulin-F	AGGCTTTCTTGCATTGGTACACGC	
β-tubulin-R	TATTCGCCTTCTTCCTCATCGGCA	
PoMAC1a-OE-F	GGTCAAAGTTACTAGTATGGTTCTGGTCGACAACA	Construction of OE plasmids
PoMAC1a-OE-R	CAATTCTAGAGGGCCCTTATCGGAAAGTATCCCTGAATGG T	
PoMAC1b-OE-F	GGTCAAAGTTACTAGTATGGTCTTCGTCAATAACAAGAAG T	
PoMAC1b-OE-R	CAATTCTAGAGGGCCCTCAGCGGCCGGCGCAGCA	
PoMAC1a-sence-F1	CCATCTCCTCAGATCATGGTTCTGGTCGACAACAA	Construction of RNAi plasmids
PoMAC1a-sence-R1	TAAGCTCTAAACTAGGTGATGAACGGGGCCTGC	
PoMAC1a-anti -F2	CGTTCATCACACTAGGAGAACGGGGCGTAGTTCC	
PoMAC1a-anti-R2	CAATTCTAGAGGGCCATGGTTCTGGTCGACAACA	
PoMAC1b-sence-F1	CCATCTCCTCAGATCATGGTCTTCGTCAATAACAAGAAG	
PoMAC1b-sence-R1	TAAGCTCTAAACTAGCGAGAAGATAGGAGGAAGTTCAAG	
PoMAC1b-anti-F2	TATCTTCTCGACTAGCGAGAAGATAGGAGGAAGTTCAAG	
PoMAC1b-anti-R2	CAATTCTAGAGGGCCATGGTCTTCGTCAATAACAAGAAG	
hyg-F	TATTCCTTTGCCCTCGGACG	Detection of transformants
hyg-R	ATGAAAAAGCCTGAACTCACC	

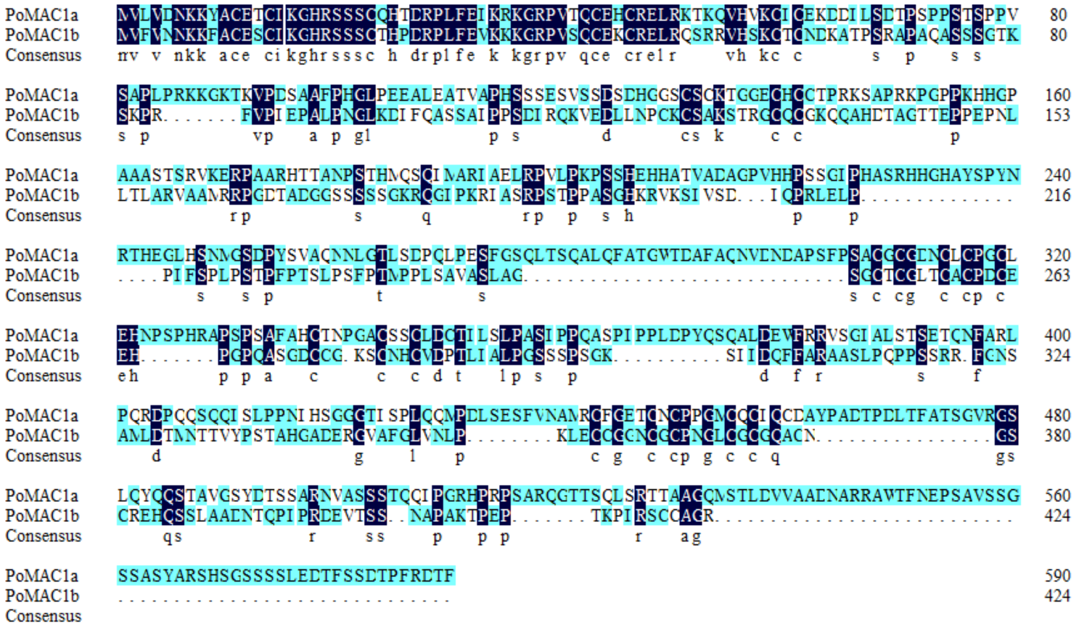

Figure S1. Amino acid sequence alignment of PoMAC1a and PoMAC1b.

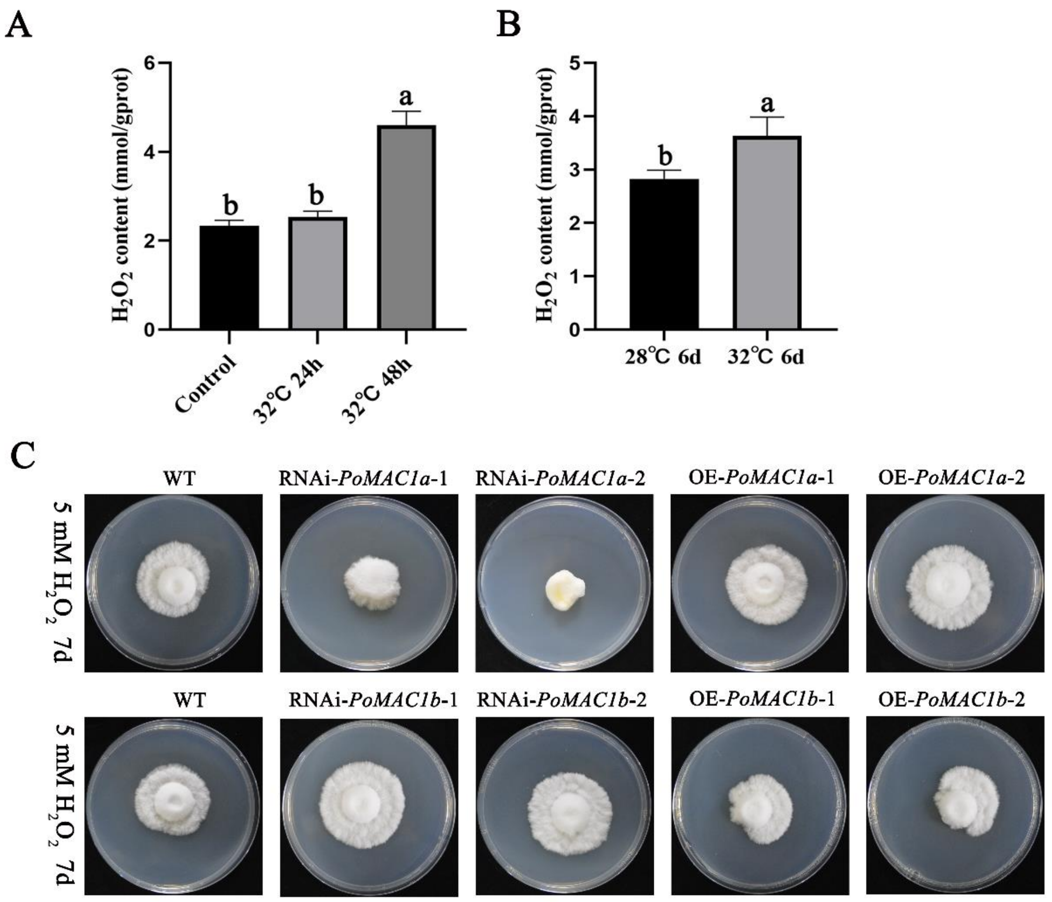

Figure S2. Detection of H_2O_2 tolerance of the tested strains. (A) Detection of H_2O_2 content after 32 °C stress for different times (24 h, 48 h). (B) The H_2O_2 content after cultivation 6 days at 32 °C and 28 °C. (C) Detection of tolerance of different mutant strains to exogenous H_2O_2.
